# The Power of Passion in Entrepreneurship Education: Entrepreneurial Role Models Encourage Passion?

**Published:** 2017-07

**Authors:** Katharina Fellnhofer

**Affiliations:** Lappeenranta University of Technology

**Keywords:** Authoring tools and methods, computer-mediated communication, cross-cultural projects, interdisciplinary project, multimedia/hypermedia system

## Abstract

This study of Entrepreneurship Education (EE) centers on the impact of entrepreneurial role models on entrepreneurial passion, which also is expected to influence entrepreneurial intention. Based on 426 individuals recruited primarily from Austria, Finland, and Greece, Structural Equation Modeling (SEM) reveals the significant direct and indirect effects of entrepreneurial role models on entrepreneurial intention, mediated by entrepreneurial passion. These effects were found to be stronger following multimedia presentation of entrepreneurial stories, confirming the fruitful spillover effects of the innovative educational use of computers on entrepreneurial intentions among nascent entrepreneurs. Drawing on the theory of planned behavior (TPB) and social learning theory, this study confirms both the positive impact of entrepreneurial role models and significant short-term effects of web-based multimedia in the context of EE. This narrative approach is shown to be an effective pedagogical instrument in enhancing individual orientation toward entrepreneurship to facilitate entrepreneurial intention. This study identifies the great potential of these pioneering methods and tools, both for further research in the academic community and for entrepreneurship educators who hope to promote entrepreneurial intention in aspiring entrepreneurs. The findings are also relevant for policy makers designing effective instruments to achieve long-term goals.

## Introduction

Although research on entrepreneurship education (EE) is burgeoning across the globe (e.g., Fayolle, Gailly & Lassas-Clerc, 2006; Souitaris, Zerbinati & Al-Laham, 2007), innovative contributions using multimedia (e.g., Béchard & Grégoire, 2005) and inspirational storytelling (e.g., Godsey & Sebora, 2009) have been relatively neglected in this field. While EE literature reviews by Lorz, Mueller & Volery (2013) and Mason and Siqueira (2014) have highlighted how the impact of different pedagogical instruments varies across studies on EE, evaluations of entrepreneurial educational initiatives remain inconsistent (Bae, Qian, Miao & Fiet, 2014; Lorz, Mueller & Volery, 2013; Rideout & Gray, 2013). Several such assessments have indicated the positive impact of EE courses (e.g., Fayolle, Gailly & Lassas-Clerc, 2006; Peterman & Kennedy, 2003; Souitaris, Zerbinati & Al-Laham, 2007), but other researchers such as Oosterbeek, van Praag, and Ijsselstein (2010) emphasize their lack of significant effects. For this reason, there is a need for more research concentrating on impactful pedagogical designs.

Narratives and storytelling can inspire, engage, and encourage through the articulation of knowledge and the cooperative sharing of and reflection on experiences between individuals. However, though research on EE literature has yet to consider the impact of entrepreneurs presenting their stories via multimedia. Few studies have addressed the use of narratives in EE (e.g., Fletcher & Watson, 2007; Godsey & Sebora, 2009; Harmeling & Sarasvathy, 2013). There is evidence that building an entrepreneurial identity is a key element in becoming a successful entrepreneur, and that this identity is developed through inspiration and engagement with peers (Falck, Heblich & Luedemann, 2012; Obschonka, Goethner, Silbereisen & Cantner, 2012). Engagement with entrepreneurs’ experiences improves legitimacy (Clarke, 2011; Essers & Benschop, 2007; Wry, Lounsbury & Glynn, 2011), and these experiences can be communicated in EE through a narrative or storytelling approach (Boje & Smith, 2010; Down & Warren, 2008; Fletcher & Watson, 2007; Johansson, 2004; Linstead & Hytti, 2005; Matlay & Harmeling, 2011; Rae, 2005). Verbal interaction and collaboration in the course of EE initiatives facilitates the development of an entrepreneurial identity (Donnellon, Ollila & Middleton, 2014). However, the impact of web-based multimedia entrepreneurial stories has not been evaluated, which is the primary aim of the present study.

More than ten years ago, Kuratko’s (2003, 2005) contributions on the development, trends, and challenges in EE stressed the importance of innovation-driven approaches and designs. Kuratko emphasized that progress in this area depended on continuously expanding the range of applied tactics and pedagogies. In line with the recommendations of Kuratko and other EE authors (e.g., Solomon, Duffy & Tarabishy, 2002; Lautenschläger & Haase, 2011), as well as the European Commission (2013), this study assesses the impact of promoting entrepreneurial stories using the narrative storytelling method, in which successful entrepreneurs, via multimedia, offer inspiring insights into their past and present.

Prior research has demonstrated that opinions and behaviors communicated by others can influence an individual’s (potential) entrepreneurial decisions (Ajzen, 1991; Akerlof & Kranton, 2000). Having a role model can significantly stimulate one’s career ambitions (Douglas & Shepherd, 2002; Krueger, Reilly & Carsrud, 2000; Scherer, Adams & Wiebe, 1989; Scherer, Adams, Carley & Wiebe, 1989). A role model is an individual who has the ability to inspire, stimulate, and encourage others to engage in a given activity in this case, entrepreneurship (Shapiro, 1987; Wright, Wong & Newill, 1997). Evidence suggests approximately 35%–70% of entrepreneurs were influenced by entrepreneurial role models when starting their ventures (Scherer, Adams & Wiebe, 1989). For present purposes, we build on two fundamental theories: the theory of planned behavior (TPB) as initially proposed by Ajzen (1991) and social learning theory (Bandura, 1977; 1986), emphasizing the impact of observing others on behavior. Thus, this study also builds on Fishbein and Ajzen’s (1977) theory of reasoned action, which was the predecessor to TPB.

For several decades, researchers in entrepreneurship have explored passion and its influence (Baum, Locke & Smith, 2001; 2004; Breugst, Domurath, Patzelt & Klaukien, 2012; Cardon, 2008; Cardon, Gregoire, Stevens & Patel, 2013; Cardon & Kirk, 2015; Shane, Locke & Collins, 2003), stressing the positive role of passion in higher-level entrepreneurial effort and growth (Baum, Locke & Smith, 2001; Drnovsek, Cardon & Patel, 2016; Murnieks, Mosakowski & Cardon, 2014). However, although the influence of entrepreneurial role models has been assumed, scholars such as Gibson (2004) and Bosma, Hessels, Schutjens, Van Praag, and Verheul (2012) have stressed the need for more research in this area. On that basis, the present study uses a pretest-posttest research design to explore whether and how multimedia, as an EE teaching device, can convey the contagious passion of entrepreneurial role models. Based on prior studies of the use of computers in EE (e.g., Chang & Lee, 2013; Drent & Meelissen, 2008; Ngai, 2007), we expect this new multimedia approach to prove itself an instrumental teaching tool, boosting entrepreneurial intention through transferred entrepreneurial passion. By evaluating this form of multimedia storytelling in three European countries (Austria, Finland, and Greece) we will contribute to the growing discussion of the value of entrepreneurial passion in EE. These innovative perspectives on the entrepreneurial spirit in different disciplines will illuminate and enrich the multi-faceted domain of EE. The following research question is addressed:

“Do entrepreneurial role models have a positive impact on entrepreneurial intention mediated by entrepreneurial passion in a web- and computer-based educational setting?”

This study centers on the impact of entrepreneurial role models on entrepreneurial passion, which also is expected to influence entrepreneurial intention. Based on 426 individuals, results reveal the significant direct and indirect effects of entrepreneurial role models on entrepreneurial intention, mediated by entrepreneurial passion. Drawing on the TPB and social learning theory, this study confirms both the positive impact of entrepreneurial role models and significant short-term effects of web-based multimedia in the context of EE. This narrative approach is shown to be an effective pedagogical instrument in enhancing individual orientation toward entrepreneurship to facilitate entrepreneurial intention. This study identifies the great potential of these pioneering methods and tools, both for further research in the academic community and for entrepreneurship educators who hope to promote entrepreneurial intention in aspiring entrepreneurs. The findings are also relevant for policy makers designing effective instruments to achieve long-term goals.

Following this research motivation, the theoretical background and developing hypotheses in relation to entrepreneurial role models, entrepreneurial passion and entrepreneurial intention are outlined. Next, we discuss the applied methodologies, research settings and sample characteristics, data reliability and validity, and control variables of the research model. Discussion of the results and analysis is followed by a summary of key findings, limitations, and implications, concluding with recommendations for future research.

## Theoretical Background and Hypothesis Development

### Entrepreneurial Role Model Exposure

Entrepreneurs can be characterized as individuals who discover and experience business opportunities (Baum & Locke, 2004). Within this multi-faceted, multi-functional, and multidisciplinary context, entrepreneurial efforts are realized mainly in the creation of new ventures (Baron, 2008; Venkataraman, 1997). A role model is someone who sets an example and encourages others to make certain career path choices or to pursue certain objectives (Basow & Howe, 1980; Shapiro, Haseltine & Rowe, 1978). As such, role models play an influential role in guiding an individual’s career path, especially within occupational frameworks (Krumboltz, Mitchell & Jones, 1976) such as entrepreneurship. Parental role models for entrepreneurs have been intensively discussed (e.g., Chlosta, Patzelt, Klein & Dormann, 2012; McDougall, Robinson & DeNisi, 1992), as has the influence of networks (Fernández-Pérez, García-Morales & Pullés, 2016; Lerner, Brush & Hisrich, 1997) and peer groups (Falck, Heblich & Luedemann, 2012; Giannetti & Simonov, 2009; Koellinger, Minniti & Schade, 2007; Nanda & Sørensen, 2010; Stuart & Ding, 2006). Davidsson and Wiklund (1997) reported that an awareness of other entrepreneurs boosts entrepreneurial ambitions and activities. Yet although it is widely acknowledged that individual behavior tends to be influenced by identity (e.g., Ajzen, 1991; Akerlof & Kranton, 2000), Bosma, Hessels, Schutjens, Van Praag, and Verheul (2012) noted that empirical research on the specific characteristics and importance of entrepreneurial role models is relatively scarce.

Gibson (2004) connected role models to the theoretical constructs of role and willingness to identify with others, and to the modeling of mental skills and forms of behavior between two individuals, indicating that role models tend to be perceived as similar to oneself. The power of role models can also be illuminated by identification and social learning theory (Gibson, 2004). Identification with role models helps individuals to define their self-concept (Akerlof & Kranton, 2000), and according to social learning theory (Bandura, 1977; 1986), individuals are fascinated by role models who encourage their development (Gibson, 2004). In line with this argument, Nauta and Kokaly (2001) found that entrepreneurial role models provide both inspiration and guidance (e.g., Bosma, Hessels, Schutjens, Van Praag & Verheul, 2012). These studies build on the Penrosian resource-based view, linking entrepreneurship with human, financial, and social capital (Davidsson & Honig, 2003; Parker & Van Praag, 2006).

While research suggests a link between available role models and the choice of entrepreneurship as an attractive career path, in-depth relationships in the context of EE have not yet been studied. The precise nature of the impact of role models on entrepreneurial intention remains unclear, and a better understanding of this phenomenon is important in light of the potential of new ventures for economic growth and innovation (Van Praag & Versloot, 2007).

Synthesizing the theories of role identification, social learning and role models’ power as discussed by Gibson (2004) and Nauta and Kokaly (2001), the present study argues that entrepreneurial role models have the power to (1) directly transfer their entrepreneurial passion to nascent entrepreneurs within a web- and computer-based educational setting, which will (2) influence perceived entrepreneurial intention, and (3) result in greater direct and indirect effects underpinning the short-term effectiveness of entrepreneurial stories in the EE context. By exploiting multimedia entrepreneurial stories, the assumed interrelationship between entrepreneurial role models, entrepreneurial passion, and entrepreneurial intention is tested here using a pretest-posttest design structured around a series of hypotheses.

### The Direct Effects of Entrepreneurial Role Models on Passion

“If you’re passionate about something and you work hard, then I think you will be successful.”

— Pierre Omidyar, founder and chairman of eBay

“If you just work on stuff that you like and you’re passionate about, you don’t have to have a master plan with how things will play out.”

— Mark Zuckerberg, founder and CEO of Facebook

As these quotes suggest, many successful entrepreneurs are convinced that passion was a key ingredient in their business success. Vallerand (2010, p. 97) defined passion as *“a strong inclination toward a self-defining activity that people love, find important, and in which they invest time and energy*.” Passion can motivate someone to pursue an activity (Vallerand et al., 2003). While Vallerand et al. (2007, 2008) have investigated passion across various disciplines, entrepreneurship scholars have identified the role of passion in the entrepreneurial framework (Baum, Locke & Smith, 2001, 2004; Shane, Locke & Collins, 2003); in short, entrepreneurs’ hearts beat to the rhythm of their entrepreneurial passion.

Entrepreneurial stories offer new insights into the dynamics that foster entrepreneurship, as entrepreneurs embody entrepreneurial passion (Chen, Yao & Kotha, 2009). Early research in psychology defined passion as an element of intimate love (e.g., Baumeister & Bratslavsky, 1999; Hendrick & Hendrick, 1989; Sternberg, 1986), or as a factor in achieving objectives (e.g., Duckworth, Peterson, Matthews & Kelly, 2007). Prior research suggests that passion is integral to important entrepreneurial personality traits such as persistence (Cardon, Gregoire, Stevens & Patel, 2013; Cardon & Kirk, 2015) and contagion of positive influence (Breugst, Domurath, Patzelt & Klaukien, 2012; Cardon, 2008). It has also been argued that passion facilitates creativity and the exploration and exploitation of entrepreneurial opportunities (Baron, 2008), attracts investors to finance businesses (Hsu, Haynie, Simmons & McKelvie, 2014; Mitteness, Sudek & Cardon, 2012), and drives entrepreneurial effort and growth (Baum, Locke & Smith, 2001; Drnovsek, Cardon & Patel, 2016; Murnieks, Mosakowski & Cardon, 2014). Following this line of argument, passion might be expected to impact positively on both before and after observing entrepreneurial role models in the pedagogical sphere.

In assessing the multimedia entrepreneurial storytelling platform, we assume that the impact of passion will be stronger after observing entrepreneurial stories than before. To compare pre and post direct effects, we propose the following hypotheses.

**Table:** 

H1a	Entrepreneurial role models have a positive impact on entrepreneurial passion before watching multimedia entrepreneurial stories.
H1b	Entrepreneurial role models have a positive impact on entrepreneurial passion after watching multimedia entrepreneurial stories.

A further research question, concerning entrepreneurial passion as a prelude to entrepreneurial intention, has not yet been addressed in the literature. It is reasonable to ask whether entrepreneurial passion is in fact the key ingredient for future entrepreneurial intention. From a theoretical perspective, passion possess great power; in particular, it entails positive powerful drivers for actions that are vital to one's self-identity (Cardon, Wincent, Singh & Drnovsek, 2009; Farmer, Yao & Kung- Mcintyre, 2011; Fauchart & Gruber, 2011).

The TPB is the predominant theoretical framework grounding this formulation. The TPB holds that three separate attitudinal variables shape intention: attitude towards the behavior, subjective norms, and perceived behavioral control. Attitude reflects the degree to which someone assesses a particular behavior as encouraging or not; a subjective norm is a perceived social pressure or expectation (Ajzen, 1988); and perceived behavioral control shapes awareness of one’s ability to successfully implement a behavior (Ajzen, 1991). Merging these three perceptions into one variable, entrepreneurial intention expresses the robust commitment of an individual to become an entrepreneur by launching a new venture (Krueger & Carsrud, 1993; Zapkau, Schwens, Steinmetz & Kabst, 2015).

Numerous studies have highlighted the power of entrepreneurial intention as a key milestone for entrepreneurial activities (Bird, 1988; Krueger & Carsrud, 1993), and the literature suggests that entrepreneurial intentions are the best predictor of planned behaviors such as launching a new venture (Bagozzi, Baumgartner & Yi, 1989; Kim & Hunter, 1993; Zapkau, Schwens, Steinmetz & Kabst, 2015). In general, entrepreneurial passion is believed to facilitate entrepreneurial intention—that is, the greater the passion for the entrepreneurial behavior, the stronger the individual's intention to achieve it. In the context of EE, an adequate teaching instrument would be expected to show the encouraging effects of entrepreneurial passion on entrepreneurial intention, especially after the instrument has been implemented. In assessing the multimedia entrepreneurial storytelling platform, we compare the pre and post direct effects by means of the following hypotheses, assuming that the post effects are stronger than the pre effects.

**Table:** 

H2a	Entrepreneurial passion has a positive impact on entrepreneurial intention before watching multimedia entrepreneurial stories.
H2b	Entrepreneurial passion has a positive impact on entrepreneurial intention after watching multimedia entrepreneurial stories.

### The Mediating Effect of Passion on Entrepreneurial Intention

Social learning theory, as originally proposed by Bandura (1977, 1986), emphasizes how observing others impacts on behavior attainment. Based on this theory, observational learning from entrepreneurial role models should have the power to influence an individual’s personality development (Bandura, 1977) through entrepreneurial passion, which can be expected to shape entrepreneurial intentions. Entrepreneurial role models tend to be strong characters who are valuable to observe when revising behavior to achieve one’s goals. The investigations of Scherer, Adams, Carley, and Wiebe (1989) and Shapero and Sokol (1982) showed that children are subject to their relatives’ behaviors. These relatives appear to be important role models for the development of attitudes and values. There is evidence that a role model’s impact reflects their importance and credibility (Scherer, Adams, Carley & Wiebe, 1989; Shapero & Sokol, 1982). Therefore, one might expect that watching successful entrepreneurs will strengthen any intention that may have existed before watching them. In line with the evidence that children learn by watching their parents and adopting these behaviors, we would anticipate this effect in watching entrepreneurial stories via multimedia.

A number of well-known researchers (Bandura, 1986; Schröder & Schmitt-Rodermund, 2006), argue that observing others can affect an individual’s career choices and decisions. Offspring can be encouraged to choose a particular career path based on informal observations of their familial role models (Barling, Dupre & Hepburn, 1998; Krumboltz, Mitchell & Jones, 1976; Mitchell & Krumboltz, 1984). Therefore, we anticipate that multimedia access to entrepreneurial role models will be perceived as encouraging, with a positive attitudinal impact on those considering becoming entrepreneurs. This assumption is based on earlier studies (Krueger, 1993; Matthews & Moser, 1996; Zapkau, Schwens, Steinmetz & Kabst, 2015). It seems likely, then, that exposure to successful and passionate entrepreneurial role models will affect an individual’s intention to become an entrepreneur. According to Scherer, Adams, Carley, and Wiebe (1989), observation of role models enables individuals to learn specific skills, knowledge, and behaviors that are relevant and essential to embarking on a new venture. Earlier findings demonstrated that entrepreneurial parents can transfer informal business knowledge to their offspring (e.g., Holtz-Eakin, 2000; Scherer, Brodzinski & Wiebe, 1991); it is expected this will also hold true in the short term after exposure to entrepreneurial role models via multimedia, strengthening entrepreneurial intention.

In summary, an entrepreneurial role model watched online is expected to have a positive influence, boosting the desire to become an entrepreneur as argued in the literature on role models (Van Auken, Fry & Stephens, 2006). In other words, an entrepreneurial role model may be expected to have a positive influence on entrepreneurial intentions and ultimately on entrepreneurial activity (Krueger Jr, Reilly & Carsrud, 2000). It expected that individuals will return higher scores for entrepreneurial intention after watching entrepreneurial stories, and that this impact will be mediated by entrepreneurial passion. In assessing the multimedia entrepreneurial storytelling platform as an effective EE teaching instrument, we compare both pre and post direct and indirect effects by means of the following hypotheses.

**Table:** 

H3a	Entrepreneurial role models have a positive impact on entrepreneurial intention mediated by entrepreneurial passion before watching multimedia entrepreneurial stories.
H3a	Entrepreneurial role models have a positive impact on entrepreneurial intention mediated by entrepreneurial passion after watching multimedia entrepreneurial stories.

Relating prior entrepreneurial exposure to Ajzen's (1991) TPB and to social learning theory (Bandura, 1977, 1986), Figure 1 illustrates the comprehensive rationale for our hypotheses concerning how entrepreneurial role model exposure impacts on entrepreneurial intention, mediated by entrepreneurial passion.

## Methods

### Research Settings and Sample Characteristics

This project adopts common standards and well-accepted methodologies in social science research. The online research platform provides a multimedia toolkit for EE, available free of charge online. The seven entrepreneurs are active globally in diverse sectors that include information and communication technology (ICT), energy, consulting, transportation, financing, and production. All are founders of small and medium-sized companies located in Austria, Germany, Spain, Finland, USA, and Australia. An introductory video presents all the entrepreneurs.

As recommended by other researchers (e.g., López, Valenzuela, Nussbaum & Tsai, 2015), an adequate sample was used to provide a robust foundation for this study. The subjects fall into the age range 18–24 years, and the gender distribution is representative of a three-country-average, with almost equal group sizes in each country. Before commencing data collection, a pre-testing phase was conducted from December 2015 to February 2016 with individuals from different target groups: three Finnish students, three Austrian students, three Austrian students from vocational schools, two Austrian educators, two Finnish professors, two German-speaking professors, and two Austrian individuals not enrolled in any course. Table 1 shows the total sample for this empirical study, which involved a questionnaire-based survey, conducted from February 2016 to July 2016 in Austria, Finland, and Greece.

### Data Reliability and Validity

In this empirical investigation, several methods were applied to reduce risk of common method bias. First, anonymity and confidentiality were ensured (Reio, 2010). Intra-class correlations were employed for all items significant at the 0.001 level, indicating a strong level of interrater reliability (Jones, Johnson, Butler & Main, 1983; James, Demaree & Wolf, 1984). The applied measures are multi-item scales modified from previous studies. The five-item scale for inspiration/modeling of an entrepreneurial role model (see Table 7, Appendix) was adapted from Nauta and Kokaly (2001). Items related to entrepreneurial intention (see Table 8, Appendix) were taken from Liñán and Chen (2009) and from Kautonen, Gelderen, and Fink (2013). Items measuring entrepreneurial passion (see Table 9, Appendix) were adapted from the work of Cardon, Gregoire, Stevens, and Patel (2013) on conceptual foundations and scale validation. All measures of the three domains of inventing, founding, and developing have been merged for an overall entrepreneurial passion score. Participants indicated their level of agreement for each item from 1 (strongly disagree) to 7 (strongly agree).

To strengthen the measuring instrument, a number of reliability and validity tests were applied at different levels (Letz & Gerr, 1995). As recommended by colleagues (e.g., López, Valenzuela, Nussbaum & Tsai, 2015), a Confirmatory Factor Analysis (CFA) underpins the study’s reliability and validity. The CFA resulted in an excellent overall model fit (see section 4.1 for more detail). The Kaiser-Meyer-Olkin (KMO) measure (Dziuban & Shirkey, 1974) was used to test whether items delivered sufficient information. The KMO values are above 0.5 for all items (see Table 12, Appendix); according to Kaiser (1974), the values are all above 0.838, which indicates excellent reliability. All determinants of the correlation matrix of correlating item groups exceed the threshold of 0.00001, and all communalities are above 0.5. The model satisfactorily explains the total variance (Hair, Anderson, Tatham & Black, 1995; Lattin, Carroll & Green, 2003; Smith, Caputi & Rawstorne, 2007). Furthermore, as indicated in Table 12, all Cronbach’s alpha values are above 0.858, indicating strong internal consistency (Nunnally, 1978; Hair, Anderson, Tatham, and Black (1995); no items were omitted to achieve these values. Construct validity is significant for all variables (t > 3.1; p < 0.001), as illustrated by the standardized factor loadings (Hair, Black, Babin, Anderson & Tatham, 2010). All indicator reliabilities with values above 0.4 are sufficient, according to Bagozzi and Baumgartner (1994), and composite reliability is also acceptable, with values above 0.6 (Bagozzi 1988). Average variance extracted (AVE) for all variables is above 0.5 and therefore acceptable, as indicated by Fornell and Larcker (1981). Overall, these CFA results confirm the reliability of the research instrument.

As a final step, further validity tests were performed. In this framework, construct validity and content validity were successfully achieved (Murphy & Davidshofer, 1988; Fraenkel & Wallen, 1993). As indicated in the bivariate Pearson correlation in Appendix Tables 10 and 11, all correlations between items and variables are significant. These outcomes confirm that the questionnaire quantifies the concept it is intended to measure as established by Carmines and Zeller (1990). Because of a strong correlation between pre and post assessment, the variance inflation factor (VIF) for each independent variable was analyzed. The highest detected VIF is 1.468; because it is under 2.5 (Allison, 1999), multicollinearity is not an issue in this study. It can be concluded that common-method bias is not a critical issue in this work. The constructs are sufficiently valid and reliable. These outcomes also align with the goodness-of-fit of the model (see section 4.1).

### Control Variables

To separate outcomes for independent variables, numerous control variables were built into the structural equation modeling (SEM), controlling for demographic and background variables such as age, gender, educational level, area of study, nationality, and viewed entrepreneur. First, we controlled for respondents’ age because the opportunity costs of entrepreneurship increase with age—in other words, the probability that younger people will engage entrepreneurially is higher than for older people (Levesque & Minniti, 2006). Next, we controlled for gender, as history indicates more ventures have been founded by males than by females (Brush, 1992; Goktan & Gupta, 2013; Kelley, Singer & Herrington, 2016). Because investment in human capital can improve the return on investment from entrepreneurship (Unger, Rauch, Frese & Rosenbusch, 2011), respondent education level was also embedded as a control variable. This study also entails controls at country level. Finally, we controlled for further role modelling effects in respect of the viewed entrepreneur, given that these individuals may differ in the level of inspiration they provide to observers (e.g., Arenius & Minniti, 2005; Wagner & Sternberg, 2004; Zampetakis, Lerakis, Kafetsios & Moustakis, 2015).

## Results

Table 2 presents construct means, standard deviations (SD) and correlations. As none of the correlations exceeds 0.7, there is no significant risk of multicollinearity (Anderson, Sweeney, & Williams, 2002). As expected, there is a strong correlation above 0.7 between pre and post assessment; therefore, the VIF for each independent variable was analyzed. The highest detected VIF is 1.468; because it is under 2.5 (Allison, 1999), multicollinearity is not an issue.

As indicated in Tables 3a and 3b, paired samples testing indicates that in post assessment related to the various entrepreneurial role models presented via multimedia, there is no significant difference in perceptions (F = 1.642, n.s.). However, there is a significant difference between watched entrepreneurs at the 0.001 level at both assessment levels (pre and post) with regard to entrepreneurial intention (pre: F = 4.743, *p* < 0.001; post: F = 5.807, *p* < 0.001). Overall, while entrepreneurial passion tends to decrease after watching entrepreneurial stories, entrepreneurial intention increases on average. As indicated in Tables 3a and 3b, the ANOVA analysis indicates that this short-term fall in entrepreneurial passion is similar across watched entrepreneurs. In other words, on average, watching different entrepreneurial stories decreases entrepreneurial passion but increases entrepreneurial intention. This increase in entrepreneurial intention differs significantly across watched entrepreneurs. Because this difference in entrepreneurial intention is equally significant pre and post, it can be ignored when analyzing the direct and indirect effects on entrepreneurial intention in both assessments separately.

Following the recommendations of Anderson and Gerbing (1988), SEM was applied to test the hypotheses, and CFA was used to assess the validity and reliability of the constructs.

### Goodness-of-Fit of the Measurement Model

In line with recommendations (e.g. López, Valenzuela, Nussbaum & Tsai, 2015), both the model tested and the fit indices are reported (Schreiber, Nora, Stage, Barlow & King, 2006). Table 4 indicates the criteria used to confirm acceptance of the model. Goodness-of-fit indices include chi-square, normed chi-square, goodness-of-fit index (GFI), comparative fit index (CFI), Tucker-Lewis coefficient (TLI), incremental fit index (IFI), and root mean square error of approximation (RMSEA). The chi-square value is 1355.28 for model pre (a) and 1332.71 for model post (b); chi-square/df is 2.818 for model pre (N = 426, df = 481, p-value = 0.00) and 2.782 for model post (N = 426, df = 479, p-value = 0.00). The value is between 2.0 and 5.0, indicating a moderately acceptable fit level (Hair, Black, Babin, Anderson & Tatham, 2010). Moreover, GFI is above 0.8 (to be precise, 0.835 for model pre and 0.845 for model post), which also represents an acceptable fit. CFI values (0.916 for model pre and 0.927 for model post) are also adequate because they are higher than 0.90 (Hu & Bentler, 1999; Byrne, 1994). TLI values are 0.908 for model pre and 0.920 for model post. The IFI value of 0.916 for model pre and 0.928 for model post again indicate an acceptable fit (Bentler & Bonett, 1980; Mulaik et al., 1989). Finally, RMSEA is 0.065 for both models (pre and post), which is less than 0.07 and is therefore considered acceptable (Browne & Cudeck, 1993; Rigdon 1996). Overall, then, Table 4 validates the goodness-of-fit indices for the models.

### Structural Equation Modeling

The impact of exposure to entrepreneurial role models on entrepreneurial intention as mediated by entrepreneurial passion is tested by comparing models pre and post exposure to the entrepreneurial stories. As shown in Table 5, hypotheses 1a and 1b and hypotheses 2a and 2b are confirmed, with a positive significant impact of entrepreneurial role models on entrepreneurial intention via the mediating variable entrepreneurial passion. Hypothesis 1a (that entrepreneurial role models have an encouraging impact on entrepreneurial passion before watching multimedia entrepreneurial stories), is supported by a significant positive effect expressed by Standardized Regression Weights (SRW) (SRW = 0.449, *p* < 0.001). This study demonstrates the independent variable entrepreneurial role model explains 20.1% of the variation in entrepreneurial passion before watching multimedia entrepreneurial stories. Hypothesis 1b (that entrepreneurial role models have an encouraging impact on entrepreneurial passion after watching multimedia entrepreneurial stories) can also be accepted, as we have found a significant positive influence (SRW = 0.557, *p* < 0.001). In particular, the independent variable entrepreneurial role model explains 31.0% of the variation in entrepreneurial passion before watching multimedia entrepreneurial stories. The post effect is higher comparing pre and post effects, which supports the proposition that multimedia entrepreneurial stories represent an adequate instrument.

For hypothesis 2a (that entrepreneurial passion has a positive influence on entrepreneurial intention before watching multimedia entrepreneurial stories), a significant positive influence was detected (SRW = 0.543, *p* < 0.001). The independent variable entrepreneurial passion explains 34.4% of the variation of entrepreneurial intention before watching multimedia entrepreneurial stories. In addition, prior participation in EE has a significant positive influence on intention in the pre model (SRW = 0.179, *p* < 0.001). No other control variable shows a significant influence on intention. Additionally, hypothesis 2b (that entrepreneurial passion has a positive effect on entrepreneurial intention after watching multimedia entrepreneurial stories) shows a significant impact (SRW = 0.638, *p* < 0.001). In particular, the independent variable entrepreneurial passion explains 44.7% of the variation in entrepreneurial intention after watching multimedia entrepreneurial stories. Additionally, in the post model, prior participation in EE also has a significant positive influence on intention (SRW = 0.149, *p* < 0.001). This is confirmed by an independent t-test (F = 0.574, *p* < 0.01). Gender also plays a significant role (SRW = 0.109, *p* < 0.01). While there is no significant difference between females and male in relation to perceptions of entrepreneurial role models and passion, there is a significant difference in entrepreneurial intention (F = 0.262, *p* < 0.001), confirmed by an independent t-test. In general, males (mean = 3.732) show a higher perceived level of intention than females (mean = 3.211). These significant differences in both prior participation in EE and gender must be taken into account in the design of EE. Comparing the results of hypotheses 2a and 2b, the stronger impact in 2b underlines the multimedia tool’s adequacy as a pedagogical instrument for EE. However, based on perceived entrepreneurial intention, it appears that this tool is more effective for males than for females, and for individuals who have already participated in any form of EE.

Table 5 highlights the positive significant impact of entrepreneurial role models on entrepreneurial intention via the mediating variable entrepreneurial passion. According to SEM, there was significant support for hypotheses 1a, 1b, 2a, and 2b in the mediation analysis. As an essential prerequisite, both the entrepreneurial role model in the pre model (0.449, *p* < 0.001) and the entrepreneurial role model in the post model (0.557, *p* < 0.001) are positively linked to entrepreneurial passion, supporting H1a and H1b. Furthermore, entrepreneurial passion is positively associated with entrepreneurial intention both pre (0.543, *p* < 0.001) and post (0.638, *p* < 0.001) watching the entrepreneurial stories, which supports H2a and H2b. As evidenced by this study, the entrepreneurial role model is a significant antecedent of entrepreneurial intention via entrepreneurial passion in both models (pre and post). The effects are stronger after watching the entrepreneurial videos, confirming the effectiveness of the EE instrument. While the essential proposition—that entrepreneurial role models influence entrepreneurial passion—is confirmed, the positive influence of entrepreneurial passion on entrepreneurial intention is also significantly supported by both models. Additionally, the adjusted R squares explain the proportion of variance in the dependent variables by the impact of the independent variable including the control variables. As noted above, the adjusted R squares generally range between 20.1 % and 44.7 %, indicating strong predictive value. As evidenced by this study, there is sufficient support for both hypotheses 1 and 2.

Next, the results for direct and indirect effects are significant using Monte Carlo bootstrap analyses to expose the proposed mediation. Table 6 shows outcomes for the pre and post models, providing vital support for the mediating effect. In line with hypothesis 3, the indirect effect of the entrepreneurial role model on entrepreneurial intention via entrepreneurial passion in H3a is significant (0.233, p < 0.01) in the pre model. The indirect effect of the entrepreneurial role model on entrepreneurial intention in H3b is also significant (0.319, p < 0.01) in the post model. Overall, the total effects of entrepreneurial passion (0.346 and 0.673) in the pre model are lower than the total effects of entrepreneurial passion (0.414 and 0.770) in the post model. These results support our assumption that observing the entrepreneurial role models via multimedia entrepreneurial stories further increases entrepreneurial intention.

**Figure 2:**
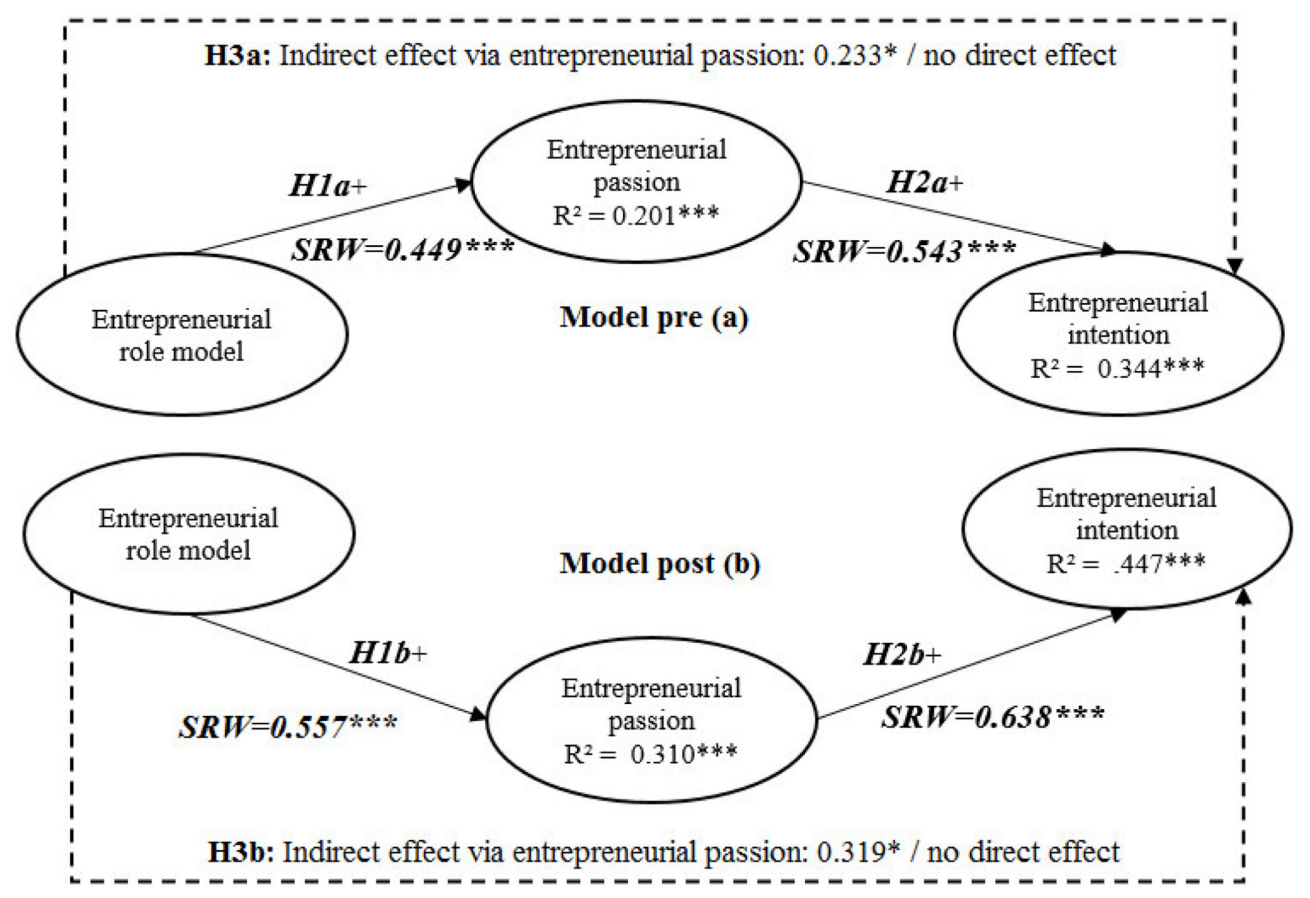
Sem Model Results: Standardized Regression Weights for the Hypotheses (N = 426) Notes: Standardized Regression Weights (SRW), 90% confidence interval. Significance codes: ***=p<.01, **=p<.05, *=p<.1. The goodness of fit indices for model pre (a): χ2= 1355.28; χ2/df = 2.818; GFI = .835; CFI = .916; TLI = .916; IFI = .908; RMSEA = .065. Goodness of fit indices for model post (b): χ2= 1332.71; χ2/df = 2.782; GFI = .845; CFI = .927; TLI = .920; IFI = .928; RMSEA = .065.

While the correlation analyses highlight the strong connection between variables, the SEM and Monte Carlo bootstrap analyses confirm the significant mediating role of entrepreneurial passion in both models. These analyses mirror the power of entrepreneurial passion as mediator pre and post watching entrepreneurial stories; overall, entrepreneurial role models show a strong positive impact on entrepreneurial intention in both models, which is even stronger following the experiment. Figure 2 illustrates these outcomes.

## Discussion

The primary purpose of this study was to unravel the impact of exposure to entrepreneurial role models on entrepreneurial intention mediated by entrepreneurial passion in a web- and computer-based educational setting. To this end, two models (pre and post exposure) were analyzed to explore these direct and indirect effects. The stability of the results across different entrepreneurial role models and target groups indicates the comprehensive generalizability of the findings.

The present study builds on previous work, including that of Kolvereid (1996) and Tkachev and Kolvereid (1999), which confirmed that TPB can explain entrepreneurial intention. Additionally, this study draws on social learning theory (Bandura, 1977; 1986) to establish that observing entrepreneurial role models has a significant impact on behavior. The findings provide empirical support for the significant positive influence of exposure to entrepreneurial role models on entrepreneurial intention mediated by entrepreneurial passion. This outcome aligns with the TPB (Ajzen 1991) which hypothesized that exogenous influences are mediated by attitudes and subjective norms (in this case, entrepreneurial passion).

In line with Bandura (1977) and Latham and Saari (1979), this study found that exposure to entrepreneurial role models using innovative techniques such as web-based channels exert a significant positive influence on entrepreneurial intention. This finding may explain why individuals experience entrepreneurial exposure as encouraging or discouraging, as discussed by Krueger (1993). While this exposure positively affects entrepreneurial intention, it has a negative influence on entrepreneurial passion. A possible explanation may be that the individual feels his or her own passion is lower compared with the passion of a successful entrepreneur. In summary, exposure to successful entrepreneurial role models positively influences entrepreneurial intention while its effect on entrepreneurial passion is more negative.

Contrary to the doubts expressed by Zapkau, Schwens, Steinmetz, and Kabst (2015), who argued that entrepreneurial role models do not lead to higher perceived behavioral control with regard to new venture creation, we found strong evidence that entrepreneurial role model exposure using multimedia techniques increases entrepreneurial intention. Our findings also support the positive influence of observational learning from role models as proposed by Scherer, Adams, Carley, and Wiebe (1989, 1989). However, we agree with Zapkau’s (2015) suggestion of a need to control for effects related to the industry specificity of role model relationships, especially where significant differences in entrepreneurial intention are detected before and after exposure to different entrepreneurs. There is evidence that the particular information transferred from entrepreneurial role models to potential entrepreneurs appears to be industry-specific (Kim, Aldrich & Keister, 2006; Zapkau, Schwens, Steinmetz & Kabst, 2015) therefore, it may not be equally useful or inspiring.

## Conclusion

Given that role models are increasingly seen as key motivators, the exploration of role models’ passion and its influence on nascent entrepreneurs is expanding in both psychology and entrepreneurship but remains in its infancy in EE. To extend the entrepreneurship literature in this regard. The central aim of this study was to identify any direct and indirect effects of entrepreneurial role models on perceived entrepreneurial intention as mediated by entrepreneurial passion in an experimental web- and computer-based educational setting. In so doing, this study also assessed the importance of multimedia for EE. The findings offer a positive answer to the primary research question: Entrepreneurial role models have a significant positive impact on entrepreneurial intention via entrepreneurial passion in a web- and computer-based educational setting. Building on the TPB and social learning theory, these results suggest that showing entrepreneurial stories via multimedia in a classroom setting can strengthen the significant positive effects on entrepreneurial intention. As such, this study is a first attempt to unravel the value for EE of role models telling their entrepreneurial stories passionately through multimedia.

### Theoretical and Practical Implications

This study’s findings contribute to both the present and future of EE in a myriad of ways. First, it takes an in-depth look at the connection between entrepreneurial passion and entrepreneurial intention created by entrepreneurial role models. Building on Ajzen’s (1991) TPB and Bandura’s (1977, 1986) social learning theory, the study extends current theory by discussing the direct and indirect impact of entrepreneurial role models on entrepreneurial passion and intention. The findings confirm that exposure to entrepreneurial stories via role models has a significant positive impact on entrepreneurial intention to start a business, and that multimedia techniques can be used to promote successful entrepreneurial role models in EE. This approach can potentially be exploited by policymakers to meet both the economic requirements of entrepreneurship and the needs of EE—an idea that has also been raised by Kolvereid and Isaksen (2006).

To empower entrepreneurial intentions, EE initiatives should also use entrepreneurial stories to stimulate entrepreneurial passion for starting a business. Entrepreneurial role model exposure can be used as a means of identifying promising entrepreneurs, and it may be useful for EE initiatives to incorporate entrepreneurial stories with other actions. In line with earlier recommendations (e.g., Van Auken, Fry & Stephens, 2006), the present findings confirm that innovative embedding of role models in EE using such techniques as multimedia storytelling can have a significant positive effect on those starting a business. However, when embedding entrepreneurial stories into the curriculum, factors such as the observed positive effects of prior participation in EE and the significant gender differences revealed by the post model have to be taken into account. Overall, it appears that multimedia observation of entrepreneurial role models has a greater effect on entrepreneurial intention among males than females and is more effective for individuals who have already participated in any form of EE. In addition, industry-specific effects of entrepreneurial role models must also be considered.

The results suggest that observation of entrepreneurial role models has a significant positive influence on entrepreneurial intention. This exposure also has the potential to influence entrepreneurial passion, in turn strengthening entrepreneurial intention. These findings also suggest that even without an entrepreneurial role model in close proximity, exposure via multimedia can positively and powerfully affect choice of entrepreneurship as a career. In this way, equal chances for a successful career path are promoted. However, entrepreneurial role model observation alone does not provide the necessary knowledge and skills to become a successful entrepreneur; instead, it serves as an additional ingredient to inspire a groundbreaking venture. These findings suggest a need for more real life elements in future EE, complemented by multimedia. In enhancing understanding of the impact of entrepreneurial role models on entrepreneurial passion and intention as potential drivers of entrepreneurship, this study will help in developing further ways to promote entrepreneurial actions and long-term outcomes, with particular regard to sustainability and effective implementation.

### Limitations

As with all academic endeavors, this study is subject to certain limitations. First, and most importantly, deploying the construct of entrepreneurial intentions as a dependent variable imposes a major limitation related to the validity and persistence of the relationship between perceived intention and actual behavior, which can only be assessed by a longitudinal approach (Davidsson & Honig, 2003). Nevertheless, as discussed in the meta- analytic review of Armitage and Conner (2001), previous research identifies the TPB as the best predictor of planned behavior across a diverse range of disciplines. In addition, participants in this study were asked to indicate their immediate career path choices in terms of their intention to become entrepreneurially active within the next twelve months. As discussed by Ajzen and Madden (1986), such a short-term time span implies a better intention-behavior relationship. Overall, as discussed in Section 3.2, the present choices show robustness, validity, and reliability in terms of prior research (e.g., Bae, Qian, Miao & Fiet, 2014; Kolvereid, 1996; Krueger, 2009; Liñán & Chen, 2009).

Another limitation is that this study’s sample comprised mainly individuals from Austria, Finland and Greece. Consequently, the findings of this investigation are dependent on cultural norms and present economic conditions in these countries and therefore may not be more widely generalizable. Nevertheless, this study serves as a point of departure for future research.

### The Potential for Future Research

The present findings suggest that future research should make further use of TPB-based models. As recommended by Cardon, Gregoire, Stevens, and Patel (2013), the three domains of passion (inventing, founding, and developing) each represent fruitful directions for future research; fine-grained examination of these specific objectives was beyond the scope of this study. This study also reiterates the suggestion by Fayolle, Liñán, and Moriano (2014) suggestion that EE research would benefit greatly from longitudinal data on the different factors in entrepreneurial intention, such as exposure to entrepreneurial role models. In this regard, validating TPB variables as significant indirect predictors of entrepreneurial behavior following multimedia exposure to entrepreneurial role models would further enrich the potential of this teaching tool. The present study needs to be repeated with larger samples and in other cultural contexts, as well as with different EE target groups. In this regard, it would be interesting to explore how students at the primary level perceive entrepreneurial role models. It would be also worth taking the time to analyze the links of the results to prior work related to entrepreneurial failure (e.g., DeTienne, Shepherd & De Castro, 2008). Finally, given the observed significant differences of entrepreneurial intention in the post model, future research should place more emphasis on how to cope with these differences to promote equality between the sexes in EE. This commitment appears crucial in light of the unexplored potential of female entrepreneurs; again, the present study serves as a useful point of departure in this regard.

## Supplementary Material

Appendix Tables

## Figures and Tables

**Figure 1:**
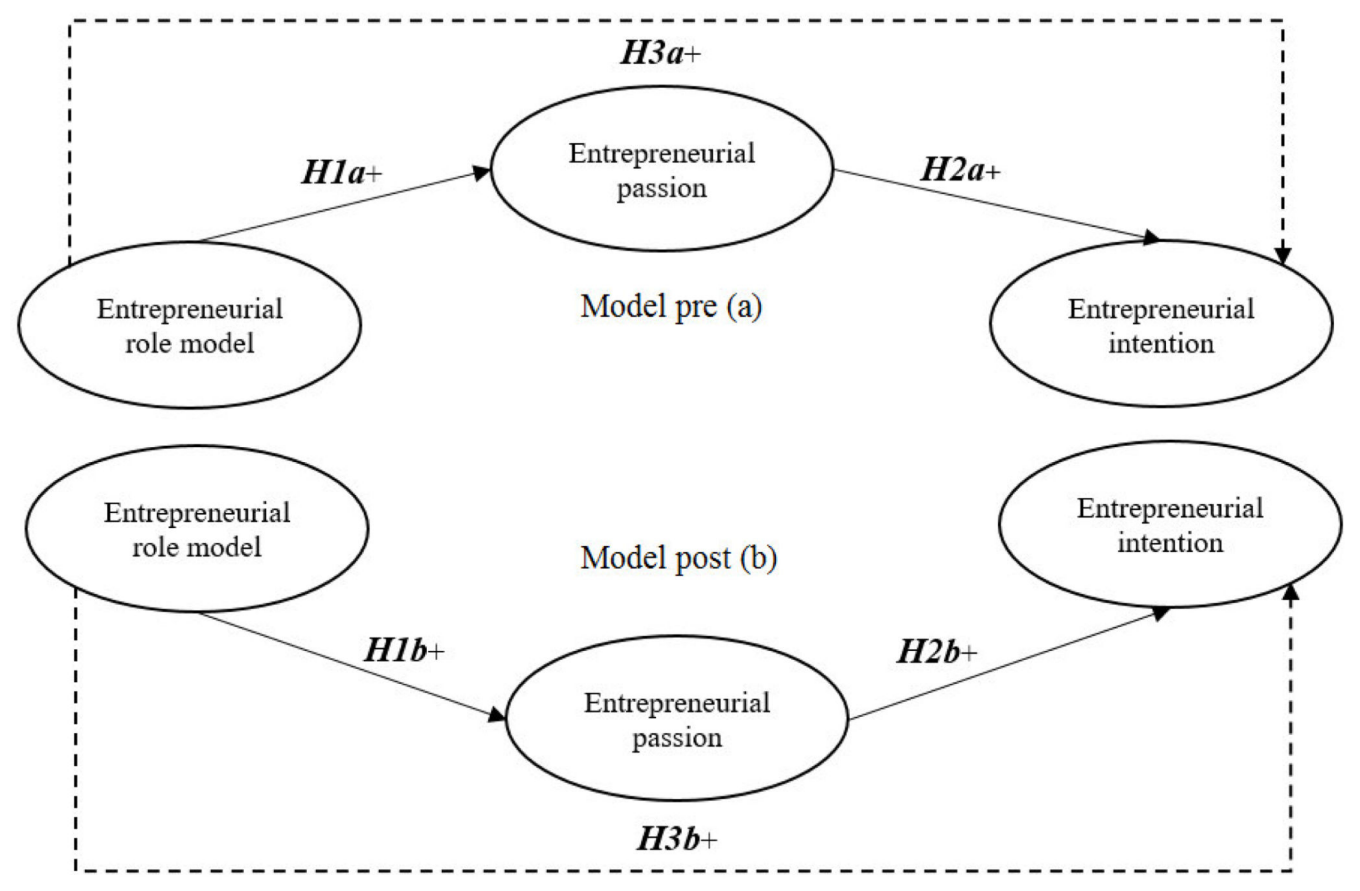
Proposed Research Model

**Table 1:** Sample Characteristics

*Entrepreneur’s business sector*	Venture Capitalist	ICT service provider	Transportation service provider	Energy producer	Tea producer and trader	Export advisor	Tax advisor
*N*	73	91	85	103	48	21	5
*Age of experiment participants*		<18	18-24	25-34	35-44	45-55	56<
	*N*	31	313	51	18	11	2
	*in %*	7.28%	73.47%	11.97%	4.23%	2.58%	0.47%

*Nationality of experiment participants*		Austria	Finland	Greece	Other		
	*N*	160	128	103	35		
		
*Gender of participants*			Female	Male	*Total*		
*Prior participation in EE*			32	81	113		
*No prior participation in EE*			127	186	313		
		
*N*			159	267			
*Total (in %)*			(37.32 %)	(62.68 %)			
		
*Population by sex (%)* *Three-country average*			45.85 %	51.7 %			

**Table 2:** Construct Means, Standard Deviations (SD), and Correlations Among Variables

		*Mean*	*SD*	*1*	*2*	*3*	*4*	*5*	*6*
*1*	PRE Entrepreneurial role model	4.02	1.31	1					
*2*	POST Entrepreneurial role model	4.04	1.33	0.641**	1				
*3*	PRE Entrepreneurial passion	4.82	1.15	0.406**	0.421**	1			
*4*	POST Entrepreneurial passion	4.67	1.20	0.331**	0.507**	0.772**	1		
*5*	PRE Entrepreneurial intention	3.40	1.42	0.305**	0.476**	0.468**	0.520**	1	
*6*	POST Entrepreneurial intention	3.54	1.46	0.287**	0.487**	0.428**	0.565**	0.857**	1

Note: n = 426; Pearson correlation (bivariate); standard deviation (SD)

** Correlation is significant at the 0.01 level (2-tailed).

**Table 3a:** Descriptives And Anova Analysis—Entrepreneurial Role Models (Pre-Assessment)

									ANOVA

*PRE ASSESSMENT*	RM 1	RM 2	RM 3	RM 4	RM 5	RM 6	RM 7	Total	Mean square between groups (within groups)
N	73	91	85	103	48	21	5	426	
*Mean*									
Entrepreneurial role model	4.24	3.84	3.80	4.17	4.10	3.74	5.04	4.02	3.34 (1.70)
Entrepreneurial passion	5.03	4.74	4.75	4.85	4.64	4.75	5.63	4.82	1.53 (1.31)
Entrepreneurial intention	3.53	2.87	3.75	3.46	3.34	3.25	5.20	3.40	9.05 (1.91)

*SD*									**F**

Entrepreneurial role model	1.06	1.31	1.51	1.18	1.34	1.43	2.12	1.31	1.959
Entrepreneurial passion	1.15	1.06	1.10	1.17	1.24	1.21	1.41	1.15	1.167
Entrepreneurial intention	1.49	1.29	1.41	1.29	1.57	1.14	1.71	1.42	4.743

*S.E.*									**Sig.**

Entrepreneurial role model	0.12	0.14	0.16	0.12	0.19	0.31	0.95	0.06	0.070*
Entrepreneurial passion	0.13	0.11	0.12	0.12	0.18	0.26	0.63	0.06	0.323
Entrepreneurial intention	0.17	0.13	0.15	0.13	0.23	0.25	0.76	0.07	0.000***

Notes: Role model (RM), standard deviation (SD), standard error (SE), significance codes: *** = p < 0.001, * = p < 0.1.

**Table 3b:** Descriptives And Anova Analysis—Entrepreneurial Role Models (Post-Assessment)

									ANOVA

*POST ASSESSMENT*	RM 1	RM 2	RM 3	RM 4	RM 5	RM 6	RM 7	Total	Mean square between groups (within groups)
N	73	91	85	103	48	21	5	426	
*Mean*									

Entrepreneurial role model	4.06	3.72	4.13	4.14	4.06	4.30	5.00	4.04	2.875 (1.751)
Entrepreneurial passion	4.85	4.60	4.47	4.79	4.45	4.85	5.45	4.67	2.293 (1.435)
Entrepreneurial intention	3.57	2.97	3.96	3.59	3.70	3.06	5.49	3.54	11.649 (2.006)

*SD*									**F**

Entrepreneurial role model	1.23	1.36	1.40	1.20	1.46	1.16	2.20	1.33	1.642
Entrepreneurial passion	1.30	1.07	1.21	1.23	1.24	0.96	1.56	1.20	1.598
Entrepreneurial intention	1.49	1.33	1.43	1.43	1.46	1.18	1.68	1.46	5.807

*S.E.*									**Sig.**

Entrepreneurial role model	0.14	0.14	0.15	0.12	0.21	0.25	0.98	0.06	0.134
Entrepreneurial passion	0.15	0.11	0.13	0.12	0.18	0.21	0.70	0.06	0.146
Entrepreneurial intention	0.17	0.14	0.16	0.14	0.21	0.26	0.75	0.07	0.000***

Note: standard deviation (SD), standard error (SE), significance codes: *** = p < 0.001.

**Table 4:** Summary of Goodness-Of-Fit Indices for the Model

Fit indices	X^2^	p-value	Chi-square/df	GFI	CFI	TLI	IFI	RMSEA
Model pre (a)	1355.28	0.00	2.818	0.835	0.916	0.908	0.916	0.065
Model post (b)	1332.71	0.00	2.782	0.845	0.927	0.920	0.928	0.065

Recommended values		>0.05	< 5	>0.80	>0.90	>0.90	>0.90	<0.07

Notes: GFI = Goodness of Fit Index, CFI = Comparative Fit Index, TLI = Tucker Lewis Index, IFI = Incremental Fit Index, RMSEA = Root Mean Square Residual.

**Table 5:** Parameter Estimates for the Model

Parameters Standardized (n = 426)	SE	SRW	t-Value (p)
Model pre			
***H1a:*** Entrepreneurial role model → Entrepreneurial passion (pre)	0.043	0.449	8.021***
***H2a:*** Entrepreneurial passion → Intention (pre)	0.071	0.543	9.437***
Model post
***H1b:*** Entrepreneurial role model → Entrepreneurial passion (post)	0.041	0.557	10.207***
***H2b:*** Entrepreneurial passion → Intention (post)	0.072	0.638	10.674***

Note: Standard error (SE), standardized regression weights (SRW), significance codes: *** = p < 0.001, ** = p < 0.05, * = p < 0.01.

**Table 6:** Direct and Indirect Effects

Hypothesis	Direct effects	Indirect effects	Total effects	S.E.	Mean	p-value
*Model pre (a)*						

H1a: Entrepreneurial role model → entrepreneurial passion (pre)	0.346	0	0.346			
H2a: Entrepreneurial passion → intention (pre)	0.673	0	0.673			
H3a: Entrepreneurial role model → intention (pre) *(Mediator: entrepreneurial passion)*	0	.233	0.233	0.044	0.231	0.233*

*Model post (b)*						

H1b: Entrepreneurial role model → entrepreneurial passion (post)	0.414	0	0.414			
H2b: Entrepreneurial passion → intention (post)	0.770	0	0.770			
H3b: Entrepreneurial role model → intention (post) *(Mediator: entrepreneurial passion)*	0	0.319	0.319	0.049	0.313	0.319*

Note: Standard error (SE), significance codes: * = p < 0.01.
