# Supplementary material for: The Power of Passion in Entrepreneurship Education: Entrepreneurial Role Models Encourage Passion?
Source: J Entrep Educ. Author manuscript; Available in PMC 2018 Jun 4. (PMC5985942)
Supplement: Appendix Tables [file NIHMS77071-supplement-Appendix_Tables.pdf]

APPENDIX

Table-7
ENTREPRENEURIAL ROLE MODEL—INSPIRATION/MODELING
(MODIFIED FROM NAUTA & KOKALY, 2001)
LEVEL OF AGREEMENT FROM 1 (STRONGLY DISAGREE) TO 7 (STRONGLY AGREE)

Domain	Item	Mean	SD	Item total correlation	Alpha if item is deleted
IM_01_pre	There is an entrepreneurial person I am trying to be like in my career pursuits.	4.15	1.64	.717	.818
IM_02_pre	There is an entrepreneurial person particularly inspirational to me in my career path.	4.15	1.63	.771	.804
IM_03_pre	In the career path I am pursuing, there is an entrepreneurial person I admire.	4.25	1.59	.724	.817
IM_04_pre	I have a mentor in my potential entrepreneurial career field.	3.36	1.67	.528	.866
IM_05_pre	I know of an entrepreneurial person who has a career I would like to pursue.	4.18	1.69	.646	.837
IM_01_post	There is an entrepreneurial person I am trying to be like in my career pursuits.	4.03	1.62	.794	.875

IM_02_post	There is an entrepreneurial person particularly inspirational to me in my career path.	4.20	1.56	.820	.870
IM_03_post	In the career path I am pursuing, there is an entrepreneurial person I admire.	4.15	1.52	.804	.874
IM_04_post	I have a mentor in my potential entrepreneurial career field.	3.64	1.57	.642	.908
IM_05_post	I know of an entrepreneurial person who has a career I would like to pursue.	4.19	1.54	.744	.886

Table-8
ENTREPRENEURIAL INTENTION (MODIFIED FROM LIÑÁN & CHEN, 2009 AND KAUTONEN, GELDEREN & FINK, 2013) LEVEL OF AGREEMENT FROM 1 (STRONGLY DISAGREE) TO 7 (STRONGLY AGREE)

Domain	Item	Mean	SD	Item total correlation	Alpha if item is deleted
Items pre					
Intention1	I am ready to do anything to be an entrepreneur.	3.30	1.68	.752	.935
Intention2	My professional goal is to become an entrepreneur.	3.67	1.74	.769	.934
Intention3	I will make every effort to start and run my own firm.	3.67	1.66	.843	.930
Intention4	I am determined to create a firm in the future.	3.93	1.71	.824	.931
Intention5	I have very seriously thought of starting a firm.	3.92	1.80	.793	.933
Intention6	I have the firm intention to start a firm someday.	4.07	1.73	.787	.933
Intention7	I plan to take steps to start a business in the next 12 months.	2.68	1.72	.712	.938
Intention8	I intend to take steps to start a business in the next 12 months.	2.67	1.70	.731	.936
Intention9	I will try to take steps to start a business in the next 12 months.	2.70	1.73	.749	.935
Items post					
Intention1	I am ready to do anything to be an entrepreneur.	3.49	1.76	.778	.942
Intention2	My professional goal is to become an entrepreneur.	3.80	1.72	.803	.941
Intention3	I will make every effort to start and run my own firm.	3.79	1.71	.841	.939
Intention4	I am determined to create a firm in the future.	3.98	1.72	.841	.938
Intention5	I have very seriously thought of starting a firm.	4.05	1.74	.828	.939
Intention6	I have the firm intention to start a firm someday.	4.05	1.70	.808	.940
Intention7	I plan to take steps to start a business in the next 12 months.	2.89	1.80	.745	.944
Intention8	I intend to take steps to start a business in the next 12 months.	2.87	1.77	.754	.943
Intention9	I will try to take steps to start a business in the next 12 months.	2.91	1.78	.737	.944

Table-9
ENTREPRENEURIAL PASSION (CARDON, GREGOIRE, STEVENS & PATEL, 2013)
LEVEL OF AGREEMENT FROM 1 (STRONGLY DISAGREE) TO 7 (STRONGLY AGREE)

Domain	Item<br>Note: inv = inventing; fnd = founding; and dev = developing.	Mean	SD	Item total correlation	Alpha if item is deleted
Passion-inv1_pre	It is exciting to figure out new ways to solve unmet market needs that can be commercialized.	4.76	1.53	.663	.945
Passion-inv2_pre	Searching for new ideas for products and services appears enjoyable to me.	4.96	1.48	.722	.943
Passion-inv3_pre	I am motivated to figure out how to make existing products/services better.	5.01	1.34	.736	.942
Passion-inv4_pre	Scanning the environment for new opportunities really excites me.	4.87	1.37	.742	.942
Passion-inv5_pre	Inventing new solutions to problems could turn into an important part of who I am.	4.77	1.49	.705	.943
Passion-fnd1_pre	Establishing a new company seems exciting to me.	4.67	1.47	.768	.941
Passion-fnd2_pre	Owning my own company will energize me.	4.78	1.61	.703	.944
Passion-fnd3_pre	Nurturing a new business through its emerging success will be enjoyable.	4.73	1.41	.761	.942
Passion-fnd4_pre	Being the founder of a business could turn into an important part of who I am.	4.72	1.51	.732	.943
Passion-dev1_pre	I will like finding the right people to whom to market a new product/service.	4.79	1.40	.762	.942
Passion-dev2_pre	Assembling the right people to work for my business will be exciting.	4.91	1.48	.776	.941
Passion-dev3_pre	Pushing myself to make my business better motivates me.	5.11	1.49	.797	.941
Passion-dev4_pre	Nurturing and growing companies could turn into an important part of who I am.	4.54	1.46	.738	.942
Passion-inv1_post	It is exciting to figure out new ways to solve unmet market needs that can be commercialized.	4.62	1.53	.712	.951
Passion-inv2_post	Searching for new ideas for products and services appears enjoyable to me.	4.79	1.48	.769	.950
Passion-inv3_post	I am motivated to figure out how to make existing products/services better.	4.75	1.48	.741	.951
Passion-inv4_post	Scanning the environment for new opportunities really excites me.	4.73	1.42	.753	.950
Passion-inv5_post	Inventing new solutions to problems could turn into an important part of who I am.	4.69	1.48	.747	.950
Passion-fnd1_post	Establishing a new company seems exciting to me.	4.61	1.52	.778	.950
Passion-fnd2_post	Owning my own company will energize me.	4.74	1.58	.749	.950
Passion-fnd3_post	Nurturing a new business through its emerging success will be enjoyable.	4.60	1.45	.774	.950
Passion-fnd4_post	Being the founder of a business could turn into an important part of who I am.	4.55	1.50	.744	.951
Passion-dev1_post	I will like finding the right people to whom to market a new product/service.	4.61	1.48	.792	.949
Passion-dev2_post	Assembling the right people to work for my business will be exciting.	4.76	1.49	.821	.948

Passion-dev3_post	Pushing myself to make my business better motivates me.	4.78	1.56	.787	.949
Passion-dev4_post	Nurturing and growing companies could turn into an important part of who I am.	4.45	1.54	.760	.950

Table-10
BIVARIATE CORRELATION—PRE-ASSESSMENT

		1	2	3	4	5	6	7	8	9	10	11	12	13	14	15	16	17	18	19	20	21	22	23	24	25	26	27
1	IM_01	1																										
2	IM_02	,752**	1																									
3	IM_03	,614**	,698**	1																								
4	IM_04	,419**	,441**	,456**	1																							
5	IM_05	,529**	,563**	,561**	,467**	1																						
6	Passion-inv1	,239**	,214**	,329**	.074	,281**	1																					
7	Passion-inv2	,281**	,262**	,338**	,115*	,299**	,701**	1																				
8	Passion-inv3	,280**	,245**	,265**	,159**	,254**	,663**	,690**	1																			
9	Passion-inv4	,313**	,301**	,274**	,155**	,297**	,543**	,648**	,696**	1																		
10	Passion-inv5	,238**	,215**	,245**	,141**	,253**	,490**	,591**	,625**	,645**	1																	
11	Passion-find1	,296**	,279**	,234**	,141**	,284**	,478**	,506**	,528**	,583**	,546**	1																
12	Passion-find2	,334**	,298**	,243**	,113*	,246**	,364**	,431**	,461**	,524**	,470**	,783**	1															
13	Passion-find3	,242**	,255**	,253**	,160**	,261**	,486**	,512**	,535**	,591**	,587**	,680**	,700**	1														
14	Passion-find4	,301**	,281**	,291**	,188**	,276**	,417**	,479**	,433**	,487**	,537**	,669**	,687**	,684**	1													
15	Passion-dev1	,332**	,280**	,281**	,202**	,272**	,522**	,562**	,552**	,548**	,545**	,608**	,566**	,602**	,602**	1												
16	Passion-dev2	,261**	,247**	,286**	,161**	,246**	,514**	,564**	,580**	,583**	,530**	,636**	,587**	,565**	,590**	,762**	1											
17	Passion-dev3	,374**	,334**	,371**	,173**	,295**	,601**	,626**	,628**	,597**	,544**	,603**	,551**	,603**	,612**	,665**	,732**	1										
18	Passion-dev4	,319**	,265**	,290**	,199**	,278**	,530**	,511**	,541**	,545**	,560**	,563**	,509**	,577**	,676**	,608**	,626**	,691**	1									
19	Intention1	,353**	,301**	,212**	,219**	,241**	,335**	,306**	,359**	,316**	,266**	,431**	,381**	,317**	,384**	,426**	,402**	,374**	,482**	1								
20	Intention2	,348**	,298**	,224**	,252**	,241**	,358**	,315**	,366**	,335**	,247**	,450**	,424**	,375**	,398**	,395**	,413**	,413**	,446**	,796**	1							
21	Intention3	,335**	,270**	,206**	,222**	,238**	,311**	,309**	,361**	,338**	,316**	,453**	,413**	,372**	,415**	,452**	,387**	,382**	,448**	,783**	,800**	1						
22	Intention4	,286**	,273**	,196**	,216**	,227**	,339**	,347**	,401**	,345**	,337**	,502**	,473**	,405**	,445**	,412**	,434**	,422**	,412**	,672**	,757**	,810**	1					
23	Intention5	,302**	,282**	,229**	,144**	,245**	,326**	,342**	,358**	,356**	,328**	,474**	,443**	,428**	,452**	,383**	,373**	,398**	,421**	,628**	,698**	,757**	,812**	1				
24	Intention6	,282**	,258**	,193**	,167**	,216**	,282**	,302**	,347**	,348**	,297**	,480**	,431**	,380**	,419**	,343**	,347**	,385**	,358**	,619**	,679**	,739**	,802**	,826**	1			
25	Intention7	.056	.081	-.015	,200**	.082	.011	.064	.075	,108*	,120*	,228**	,193**	,174**	,153**	,164**	,118*	.046	,152**	,467**	,433**	,519**	,506**	,492**	,499**	1		
26	Intention8	.061	,103*	.001	,213**	,099*	.054	.075	,103*	,114*	,127**	,223**	,194**	,168**	,154**	,165**	,118*	.058	,167**	,484**	,460**	,530**	,522**	,501**	,514**	,948**	1	
27	Intention9	,137**	,146**	.046	,207**	,155**	,109*	,128**	,142**	,144**	,147**	,245**	,225**	,180**	,184**	,203**	,156**	,117*	,207**	,544**	,478**	,587**	,533**	,519**	,519**	,890**	,908**	1

Significance codes: *** = p < .01, ** = p < .05, * = p < .1.

Table 11
BIVARIATE CORRELATION—POST-ASSESSMENT

		1	2	3	4	5	6	7	8	9	10	11	12	13	14	15	16	17	18	19	20	21	22	23	24	25	26	27
1	IM_01	1																										
2	IM_02	,811**	1																									
3	IM_03	,737**	,766**	1																								
4	IM_04	,520**	,563**	,570**	1																							
5	IM_05	,644**	,639**	,665**	,623**	1																						
6	Passion-inv1	,337**	,332**	,335**	,160**	,275**	1																					
7	Passion-inv2	,326**	,361**	,344**	,198**	,286**	,783**	1																				
8	Passion-inv3	,387**	,396**	,379**	,222**	,346**	,735**	,762**	1																			
9	Passion-inv4	,308**	,367**	,356**	,262**	,329**	,621**	,709**	,668**	1																		
10	Passion-inv5	,308**	,322**	,361**	,262**	,328**	,566**	,592**	,628**	,645**	1																	
11	Passion-find1	,401**	,409**	,399**	,299**	,399**	,501**	,542**	,524**	,588**	,626**	1																
12	Passion-find2	,420**	,403**	,425**	,309**	,381**	,415**	,485**	,502**	,559**	,565**	,767**	1															
13	Passion-find3	,380**	,371**	,417**	,259**	,326**	,505**	,551**	,526**	,575**	,572**	,738**	,774**	1														
14	Passion-find4	,386**	,418**	,417**	,308**	,350**	,463**	,517**	,453**	,511**	,644**	,674**	,680**	,674**	1													
15	Passion-dev1	,370**	,392**	,405**	,307**	,322**	,574**	,607**	,622**	,588**	,570**	,649**	,645**	,662**	,622**	1												
16	Passion-dev2	,352**	,410**	,386**	,279**	,357**	,637**	,655**	,593**	,613**	,584**	,660**	,640**	,659**	,648**	,781**	1											
17	Passion-dev3	,377**	,383**	,414**	,271**	,311**	,587**	,657**	,618**	,608**	,566**	,609**	,633**	,631**	,585**	,683**	,720**	1										
18	Passion-dev4	,382**	,428**	,364**	,285**	,309**	,530**	,576**	,539**	,584**	,654**	,608**	,571**	,581**	,705**	,606**	,679**	,695**	1									
19	Intention1	,452**	,432**	,396**	,390**	,386**	,418**	,377**	,376**	,396**	,394**	,497**	,458**	,448**	,480**	,453**	,452**	,454**	,515**	1								
20	Intention2	,467**	,446**	,403**	,364**	,396**	,375**	,387**	,370**	,427**	,384**	,581**	,529**	,499**	,493**	,442**	,463**	,463**	,512**	,824**	1							
21	Intention3	,450**	,433**	,431**	,371**	,399**	,392**	,392**	,401**	,426**	,430**	,560**	,528**	,539**	,490**	,485**	,469**	,438**	,499**	,809**	,830**	1						
22	Intention4	,432**	,416**	,422**	,312**	,368**	,392**	,421**	,396**	,456**	,409**	,601**	,559**	,559**	,474**	,529**	,504**	,482**	,470**	,718**	,794**	,823**	1					
23	Intention5	,409**	,411**	,424**	,314**	,392**	,384**	,413**	,392**	,453**	,420**	,617**	,548**	,536**	,487**	,475**	,482**	,456**	,485**	,662**	,748**	,761**	,850**	1				
24	Intention6	,374**	,395**	,421**	,295**	,401**	,384**	,394**	,418**	,457**	,411**	,601**	,554**	,565**	,470**	,495**	,485**	,450**	,436**	,652**	,718**	,756**	,855**	,865**	1			
25	Intention7	,193**	,191**	,231**	,339**	,241**	,123*	,127**	,138**	,172**	,239**	,319**	,263**	,287**	,282**	,210**	,188**	,184**	,244**	,543**	,496**	,561**	,530**	,554**	,538**	1		
26	Intention8	,221**	,188**	,242**	,346**	,249**	,119*	,118*	,138**	,188**	,249**	,330**	,285**	,310**	,286**	,245**	,199**	,172**	,269**	,528**	,501**	,549**	,552**	,562**	,541**	,924**	1	
27	Intention9	,233**	,198**	,226**	,326**	,250**	,090	,112*	,127**	,185**	,223**	,311**	,261**	,293**	,254**	,236**	,185**	,164**	,286**	,522**	,501**	,548**	,521**	,565**	,519**	,883**	,924**	1

Significance codes: *** = p < .01, ** = p < .05, * = p <. 1.

Table-12
RESULTS OF A CONFIRMATORY FACTOR ANALYSIS (CFA)

Construct	Items	Standardized factor loadings[a]	Indicator reliability ≥0.4[b]	Composite reliability ≥0.6[c]	Cronbach's α ≥0.7[d]	AVE ≥0.5[e]	Kaiser-Meyer-Olkin Measure of Sampling Adequacy[f]	Determinant[g]	Bartlett's Test of Sphericity[h]
Inspiration/Modeling	IM_01_pre	.823	.677	.862	.858	.563	.838	.094	999.52 ***
	IM_02_pre	.880	.774						
	IM_03_pre	.789	.623						
	IM_04_pre	.537	.288						
	IM_05_pre	.672	.452						
	IM_01_post	.878	.771	.901	.904	.799	.862	.035	1410.857 ***
	IM_02_post	.904	.818						
	IM_03_post	.855	.731						
	IM_04_post	.624	.390						
	IM_05_post	.735	.540						
Perceptions of entrepreneurial passion	Passion-inv1_pre	.677	.459	.946	.947	.756	.941	.00004	4256.608 ***
	Passion-inv2_pre	.712	.508						
	Passion-inv3_pre	.749	.561						
	Passion-inv4_pre	.733	.537						
	Passion-inv5_pre	.718	.515						
	Passion-fnd1_pre	.778	.605						
	Passion-fnd2_pre	.723	.522						
	Passion-fnd3_pre	.780	.609						
	Passion-fnd4_pre	.780	.609						
	Passion-dev1_pre	.775	.601						
	Passion-dev2_pre	.790	.624						
	Passion-dev3_pre	.827	.684						
	Passion-dev4_pre	.783	.613						
	Passion-inv1_post	.692	.479	.951	.954	.774	.946	.000012	4731.97 ***
	Passion-inv2_post	.746	.557						
	Passion-inv3_post	.720	.518						
	Passion-inv4_post	.752	.565						
	Passion-inv5_post	.753	.567						
	Passion-fnd1_post	.792	.627						
	Passion-fnd2_post	.765	.586						
	Passion-fnd3_post	.781	.610						
	Passion-fnd4_post	.779	.608						
	Passion-dev1_post	.824	.679						
	Passion-dev2_post	.852	.727						
	Passion-dev3_post	.813	.662						
	Passion-dev4_post	.799	.639						
Entrepreneurial intention	Intention1_pre	.780	.609	.927	.941	.758	.899	.00003	4427.37 ***
	Intention2_pre	.836	.699						
	Intention3_pre	.902	.814						
	Intention4_pre	.905	.819						
	Intention5_pre	.850	.723						
	Intention6_pre	.832	.693						
	Intention7_pre	.549	.301						
	Intention8_pre	.566	.320						

	Intention9_pre	.600	.360						
	Intention1_post	.737	.543						
	Intention2_post	.814	.662						
	Intention3_post	.845	.714						
	Intention4_post	.933	.870						
	Intention5_post	.916	.838	.931	.947	.766	.902	.000014	4698.82 ***
	Intention6_post	.912	.831						
	Intention7_post	.578	.334						
	Intention8_post	.588	.346						
	Intention9_post	.571	.326						

[a] All factor loadings are significant ($t > 3.1$; $p < 0.001$). [b]Bagozzi & Baumgartner 1994. [c]Bagozzi 1988; Raykov (1997). [d]All Cronbach's alpha are greater than 0.7 (Nunnally, 1978; Hair, Anderson, Tatham & Black, 1995). [e]Fornell and Larcker (1981). [f]All Kaiser-Meyer-Olkin Measures of Sampling Adequacy are more than 0.5 (Kaiser, 1974). [g]All determinants of the constructs' correlation matrix are greater than the necessary value of 0.00001. [h]All significant values conclude that there are suitable correlations in the data set (Bartlett, 1937).
